# Reduction in Diarrhoea and Modulation of Intestinal Gene Expression in Pigs Allocated a Low Protein Diet without Medicinal Zinc Oxide Post-Weaning

**DOI:** 10.3390/ani12080989

**Published:** 2022-04-11

**Authors:** Julie C. Lynegaard, Niels J. Kjeldsen, Christian F. Hansen, Andrew R. Williams, Jens Peter Nielsen, Charlotte Amdi

**Affiliations:** 1 Department of Veterinary and Animal Sciences, University of Copenhagen, DK-1870 Frederiksberg C, Denmark; clr604@alumni.ku.dk (J.C.L.); arw@sund.ku.dk (A.R.W.); jpni@sund.ku.dk (J.P.N.); 2 Pig Research Centre, Danish Agriculture and Food Council, SEGES, DK-1609 Copenhagen V, Denmark; njk@seges.dk (N.J.K.); cfha@seges.dk (C.F.H.)

**Keywords:** post-weaning diarrhoea, dietary protein, medicinal zinc oxide, amino acids, antibiotics, weaning, gene expression

## Abstract

**Simple Summary:**

Post-weaning diarrhoea in pigs can be a challenge and medicinal zinc oxide can decrease the need for antibiotic treatment. Low protein diets decrease diarrhoea post-weaning and soy protein concentrate improves protein digestion when compared to a soybean meal based diet. The aim of this study was to test the effect of low protein diets with different protein sources as an alternative to medicinal zinc oxide. The study demonstrated that low protein diets can decrease diarrhoea-related antibiotic treatments but also reduces growth performance. Additionally, the study presented a difference in gut nutrient metabolism when feeding very low protein levels.

**Abstract:**

Weaning comprises a challenging period for pigs, but dietary tools can be implemented to avoid excess antibiotics usage. Therefore, we tested the effect of a 17.6% crude protein (CP) diet on growth and diarrhoea and investigated the effect of a 15.5% CP diet post-weaning on transcriptomic responses, growth, and diarrhoea-related antibiotic treatments. At weaning, pigs were divided into five dietary treatment groups in a three-phase diet from weaning to 30 kg bodyweight. The diets included a positive control group (PC) with medicinal zinc oxide, a negative control group (NC), a 17.6% CP diet based on soy protein concentrate (SP), a 17.6% CP diet based on soybean meal (SB), and a 15.5% CP diet with additional amino acids (XLA). Growth performance and the occurrence of diarrhoea were similar between the SP and SB groups. The XLA pigs had a reduced weight gain and fewer antibiotics treatments caused by diarrhoea, as well as a reduced level of blood proteins. Intestinal tissue samples from the XLA pigs displayed decreased expression of genes involved in nutrient metabolism and immune responses relative to the PC group. In conclusion, a very low CP diet reduces antibiotics treatments, but also adapts gut nutrient metabolism and reduces growth performance.

## 1. Introduction

Weaning is a critical period within pig production, where pigs are forced to adapt to nutritional, physical, and immunological changes often resulting in intestinal diseases such as post-weaning diarrhoea. Medicinal zinc oxide (ZnO) has been used as an alternative to in-feed antibiotics for many years to combat post-weaning diarrhoea in pigs [1,2,3]. However, due to environmental concerns, but also reports of an association between high doses of ZnO and an increased prevalence of antimicrobial resistant bacteria, the Committee for Medicinal Products for Veterinary Use has banned the use of ZnO from June 2022 within the EU [4]. A low level of dietary protein post-weaning (17.5 or 16.6% CP) has demonstrated to reduce post-weaning diarrhoea in pigs [5,6], and can therefore be used as a dietary tool to fight intestinal disorders, but the low protein level also results in a lower average daily gain (ADG). In addition, soybean meal is an excellent protein source for pigs as the amino acid (AA) profile complements that of grains [7]. However, soybean meal also contains several potential antinutritional factors such as trypsin inhibitors and antigenic proteins that may limit protein digestibility and growth performance in young pigs and thereby bring about an increase in incidence of diarrhoea [8,9]. Soy protein concentrate has fewer antinutritional factors and may therefore be more digestible for the newly weaned piglet [10], resulting in an improved nutrient utilization and growth [11,12]. Furthermore, inclusion of AAs are essential in low protein diets, as there may otherwise be an undersupply of essential AAs, which in turn can have a negative effect on pig health and decrease growth performance [6,13]. 

Weaning of pigs is a challenging period that activates stress and inflammation signalling pathways, and may result in irregular expression of intestinal genes and proteins [14,15,16]. RNA-sequencing uses a next-generation sequencing technology for analysis of the total gene expression [17]. There is a lack of characterisation of the pig intestinal tissues by RNA-sequencing analysis post-weaning, its relation to different dietary protein restrictions and whether intestinal gene expression could explain the positive effect on diarrhoea or the reduced weight gain in low protein diets.

The primary aim of the present study was to test the effect of a low level of dietary protein (17.6% CP) post-weaning, in diets containing different protein sources (soy protein concentrate and soybean meal) without medicinal ZnO, on the occurrence of diarrhoea and growth performance. The secondary aim was to investigate the effect of a low level of protein (15.4% CP) in the post-weaning diet without medicinal ZnO on diarrhoea treatments, growth performance, and intestinal gene expression.

## 2. Materials and Methods

The study was performed at the Danish Pig Research Centre’s experimental station in Denmark from May 2019 to January 2020, and the study complied with laws and regulations for the humane care and use of animals [18].

### 2.1. Experimental Design 

A subpopulation of 3498 weaned pigs (Duroc × (Danish Landrace × Danish Yorkshire), Danbred, Copenhagen, Denmark) from the study by Lynegaard et al. [19] were used for this study. Pigs were purchased from two Danish commercial sow herds at weaning with an entry BW between 5.5 and 9.0 kg (~28 days of age) and left the trial when they attained a BW of about 30 kg. The study was conducted as a randomised block trial, where blocks included six pens with different experimental diets. Newly weaned pigs entered the trial every week and were randomly allocated to one of five experimental diets balanced by bodyweight (small, medium, large) and gender (castrated males and females). Pigs were further divided between pens, so that within one block (six pens) there was a maximum BW variation of +/−0.25 kg per pig. 

An in-depth analysis was performed on a subgroup of 20 pigs per treatment group at days 11 and 24 post-weaning, where pigs were sacrificed for post-mortem samplings for gene expression analysis of the small intestine. 

### 2.2. Dietary Treatments

The five experimental diets included two control groups, two low protein diets with either soybean meal or soy protein concentrate as the main protein source, and a very low protein diet in a three-phase feeding programme (Table 1). The experimental diets were: standard CP levels (19.2, 18.9 and 19.1% CP) including 2,500 ppm medicinal ZnO in phase 1 (PC, positive control), standard CP levels (19.2, 18.9 and 19.1% CP) without medicinal ZnO (NC, negative control), low CP levels in Phase 1 and 2 (17.6, 17.4 and 19.1% CP) with a higher level of soy protein concentrate (SP group), low CP levels in Phase 1 and 2 (17.6, 17.4 and 19.1% CP) with a high level of soybean meal (SB group), and lastly, very low CP levels in Phase 1 and 2 (15.4, 15.1 and 19.1% CP) and supplemented with the AAs Ile, Leu, His, Phe, and Tyr (XLA, x-low protein + AA). All diets were supplemented with crystalline AA (Lys, Met, Thr, Trp, Val) to obtain an ideal AA profile. The PC and NC diets were formulated according with 2018 Danish standards for weaner feed [20], whereas the SP, SB, and XLA diets were formulated according with 2019 Danish standards [21] and based on results from a previous trial [6]. Dietary ingredients can be seen in Table 2.

### 2.3. Housing and Management Routines

Upon entry to the trial, all pigs were vaccinated with 0.5 mL Circovac^®^ (Ceva Animal Health A/S, Vejle, Denmark) against Porcine Circovirus type 2. The herd had eight sections, where four sections included 18 pens with 4 m^2^ with 10 pigs per pen, and four sections with 12 pens of 5 m^2^ with 15 pigs per pen. Each weekly batch consisted of 180 newly weaned pigs that were placed into one section. Pens had 2 m^2^ of solid floor and remaining floors were slatted. The pens had a covered part of about 40% of the floor area, and each pen had a water dispenser and an individual Spotmix feeder (Bopil A/S, Sønderborg, Denmark).

### 2.4. Feeding and Feeding System

Each pen had a Spotmix feeding system (Schauer Agrotronic GmbH, Prambachkirchen, Austria) using air-assisted transport, where pigs had ad libitum access to dry pelleted feed (Danish Agro, Sjølund, Denmark) throughout the entire trial period. Diets were based on wheat and barley and were allocated to the pigs in a three-phase feeding programme, based on both BW and age: Phase 1: BW 5.5 to 9 kg, days 1 to 11, Phase 2: BW 9 to 15 kg, ~days 12 to 27 and Phase 3: BW 15 to 30 kg, ~days 28 to 52. The feed was gradually changed over a three-day period. The change between the Phase 1 and 2 diets was set at day 11 post-weaning, to ensure that no medicinal ZnO were left in the feeder after day 14 post-weaning. 

### 2.5. Recordings and Measurements

Antibiotic treatment against diarrhoea were recorded for both individual treatments and pen-wide daily. Diarrhoea treatments with antibiotics were performed by the staff, by recognising the signs of diarrhoea disease (perineal fecal staining, sunken eyes, hollow lumbar region and unthriftiness), according to the veterinarians’ instructions. For further details about diarrhoea treatments in the trial, see Lynegaard et al. [19]. The NC group included twice as many pigs as remaining groups, as this was the reference group in Lynegaard et al. [19]. Individual BW of the subpopulation of pigs were recorded at entry (day 1), at day 11, day 24, and day 39 on a scale (Bjerringbro vægte ApS, Bjerringbro, Denmark). Pigs that required special attention in a sick pen were removed from the pen and BW was recorded. 

On day 10 and 24 post-weaning, 20 pigs from each dietary group were sacrificed. Pigs were selected within each pen, based on no visible signs of illness or injury, no antibiotic treatments from weaning to sampling, and pigs being from different pens. Pigs free from antibiotics were chosen for sampling, as antibiotics can affect the intestine positively. Pigs were euthanised by stun gun in a clean lab at the experimental station and de-bled by cutting the jugular vein. Two blood samples were collected per pig in 4 mL tubes (BD-Plymouth, Plymouth, UK) and stored at −20 °C until further analysis. 

### 2.6. Post-Mortem Examinations and Samplings

The gastrointestinal tract was removed and separated into sections (stomach, small intestine, large intestine), before weight was recorded (full and empty) on a precision scale (Radwag, Radom, Poland) and the length of the small intestine was recorded. Furthermore, digesta samples and 1 × 2 cm tissue samples were collected from the mid-jejunum, distal jejunum (approximately 75% of the jejunum length), from the ileum, and from the mid-colon (apex) into cryo-tubes with RNA-later (Invitrogen by Thermo Fischer Scientific, Denmark). Samples for gene expression were stored at −20 °C. Only blood samples and tissue samples for gene expression from the PC and the XLA group were further analysed, as we experienced the main difference between these two groups and due to financial limitations.

### 2.7. RNA-Sequencing

Total RNA was extracted from 30–40 mg of distal jejunum intestinal tissue from the PC and XLA groups at 24 days post-weaning, using a commercially available kit (miRNeasy R Mini Kit, Qiagen, CA, USA) according to the manufacturer’s guidelines. For a detailed description of the RNA extraction see Amdi et al. [22]. Libraries were prepared by polyA-selected RNA library preparation and PE100 sequenced using a DNBSEQ sequencer (BGI, Copenhagen, Denmark). Clean reads were aligned to the reference group *Sus scrofa* using HISAT and aligned reads were referenced to genes by Bowtie2 (v2.2.5) identifying 18,670 genes. Differentially expressed genes were analysed by Deseq2 and gene-set enrichment analysis was used for pathway analysis (Broad Institute: http://software.broadinstitute.org, accessed on 11 December 2020). 

### 2.8. Blood Analysis

A biochemical analysis of blood serum was performed at the Veterinary Diagnostics Laboratory (University of Copenhagen, Denmark) for albumin, protein, BASP, ALT, bilirubin, CK, cholesterol, iron, phosphate, AST, blood urea nitrogen (BUN), GGT, calcium, magnesium, sodium, and potassium (Advia 1800 Chemistry System, Siemens Healthcare Diagnostics, Tarrytown, NY, USA). This analysis was performed when blood had been collected from all pigs.

### 2.9. Statistical Analyses

Data were analysed using the statistical program R (version 1.0.153—© 2022–2017) according to the following two models with individual pigs as the experimental unit. Bodyweight and ADG were analysed using a linear mixed model (1).

(1) $$Y_{ijlm}=\mu +\alpha_{i}+\beta_{j}+\delta_{m}+\theta_{l}+\varepsilon_{ijkl}$$

where *Y_ijlm_* is the dependent variable measured (bodyweight and ADG), *μ* denotes the overall mean, *α_i_* denotes the effect of treatment (*i* = PC, NC, SP, SB, XLA), *β_j_* denotes the effect of gender (*j* = male, female), $\delta_{m}$ denotes the effect of antibiotic treatment (*m* = untreated, treated), *θ* is the random effect of block (*l* =1, 2,…., 16), and *ε_ij_* describes the random error term. 

Antibiotic treatments against diarrhoea were analysed with a logistic regression model (2).

*Y_ijl_* = *μ* + *α_i_* + *β_j_* + *θ_l_* + *ε_ijkl_* (2)

where *Y_ijl_* is the dependent variable measured (diarrhoea treatments), *μ* denotes the overall mean, *α_i_* denotes the effect of treatment (*i* = PC, NC, SP, SB, XLA), *β_j_* denotes the effect of gender (*j* = male, female), *θ* is the random effect of block (*l* =1, 2,…., 16), and *ε_ij_* describes the random error term. 

Organ and blood parameters were analysed with a linear mixed model (3).

(3) $$Y_{ijkl}=\mu +\alpha_{i}+\beta_{j}+\delta +\lambda_{k}\;+\theta_{l}+\varepsilon_{ijkl}$$

where *Y_ijkl_* is the dependent variable measured (organ and blood parameters), *μ* denotes the overall mean, *α_i_* denotes the effect of treatment (*i* = PC, NC, SP, SB, XLA), *β_j_* denotes the effect of gender (*j* = male, female), *δ* denotes bodyweight as a covariate, λ_k_ denotes the effect of day (*k* = 10, 24), *θ* is the random effect of block (*l* = 1, 2,…., 16), and *ε_ij_* describes the random error term. 

The interaction day × dietary treatment was tested and deemed not significant. The effects of gender and interactions were found to be insignificant in all analysis. Means are presented as least square means ± SEM and considered significant at *p* < 0.05.

Power calculations indicated that the chosen sample size (20 pigs per treatment group) was sufficient to detect gene expression of a fold change > 1.5 with 80% and a Type 1 error rate of less than 0.05.

## 3. Results

### 3.1. Diarrhoea Treatments

The effect of dietary treatment on the percentage of AB-treated pigs and the pigs removed from the trial at days 10, 24, and 39 post-weaning are presented in Table 3. The PC pigs had fewer diarrhoea-treated pigs throughout the entire trial period when compared with the NC pigs (PC = 43.5% AB-treated pigs vs. NC = 63.9% AB-treated pigs, *p* < 0.001). Whereas the SP and SB groups had fewer diarrhoea treatments than the NC group, but they still did not have as few AB treatments as in the PC group. Lastly, only 24.4% of the XLA pigs were AB treated at the end of the trial, which was fewer than all the other diet groups (*p* < 0.001). 

### 3.2. Growth Performance

Sex did not influence growth performance in the current trial, where castrated males at the end of the experiment at day 39 had a BW of 22.9 kg and females a BW of 22.5 kg, and both genders entered the experiment with an equal BW (6.9 kg). The effects of the experimental diets and antibiotic treatment on growth performance during the weaner period are summarised in Table 4. Antibiotic treatment had a significant effect on ADG in Phase 1 and 2, which disappeared in Phase 3 an in the overall trial period. During Phase 1, the XLA pigs had a reduced ADG (115 g/d and 95 g/d, respectively) regardless of AB treatment when compared to all other dietary treatment groups (*p* < 0.001). In the second phase, all groups had a lower ADG than the PC group (*p* < 0.001), whereas only the XLA group had a reduced ADG in Phase 3 when compared with control groups (*p* < 0.001). During the overall nursing period, the SP group had reduced ADG when compared with the PC group (*p* < 0.001), while no significant difference was detected between SP, SB, and NC pigs. Both the untreated and AB-treated XLA pigs performed poorer than pigs from all other dietary treatment groups in the overall trial period, with an overall ADG of 334.7 and 339.7 g/d, respectively, compared with an ADG of 420.9 and 427.8 g/d, respectively, in the PC pigs (*p* < 0.001). 

### 3.3. Transcriptomic Analysis

**Figure 1:**
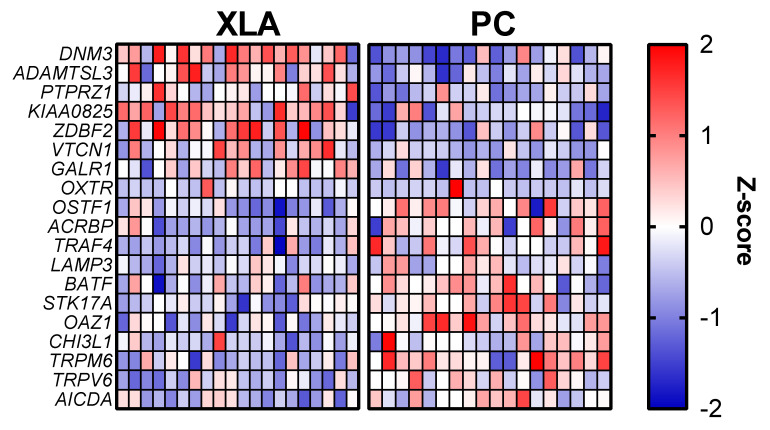
Heat map of the top down- and up-regulated differently expressed genes. The blue squares (0 to −2) are down-regulated genes and red squares (0 to +2) are up-regulated genes in XLA group when compared to the PC group. PC = Positive control with medicinal zinc oxide, XLA = Extra low protein diet with non-marked amino acids.

To characterise intestinal transcriptomic responses in pigs fed the low-protein diets, RNA-sequencing was used to identify differentially expressed genes (DEG) and transcriptional pathways. Figure 1 displays the top down- and up-regulated DEGs between the treatment groups PC and XLA (uncorrected *p*-value < 0.0001; adjusted *p*-value < 0.15). Several of the down-regulated genes (e.g., *TRPV6*, *TRPM6*, *OAZ1*, *CHI3L1*) were related to protein and/or nutrient metabolism.

**Figure 2:**
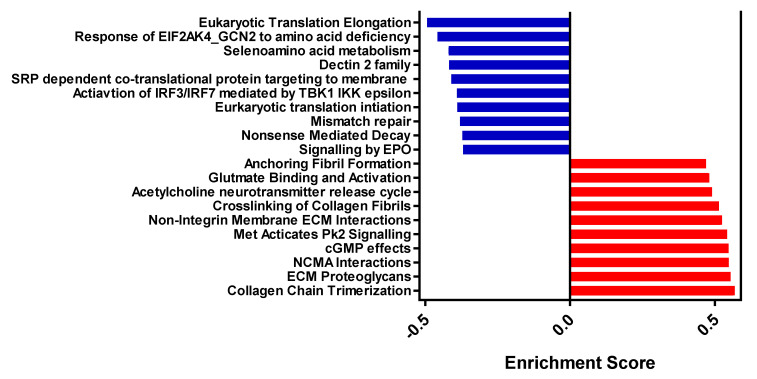
Enrichment scores of the top ten down- and up-regulated pathways in XLA group pigs relative to PC group pigs, from REACTOME database as identified by GSEA (for all pathways Q-value < 0.05). PC = Positive control with medicinal zinc oxide, XLA = Extra low protein diet with non-marked amino acids.

Gene-set enrichment analysis was performed, and 286 significantly different pathways were identified (Supplementary Tables S1 and S2). The top ten down- and up-regulated pathways are displayed in Figure 2. The down-regulated pathways were involved with protein metabolism, immune function, and translation activity, whereas the up-regulated pathways were related to extracellular matrix (ECM) organisation and collagen production.

**Figure 3:**
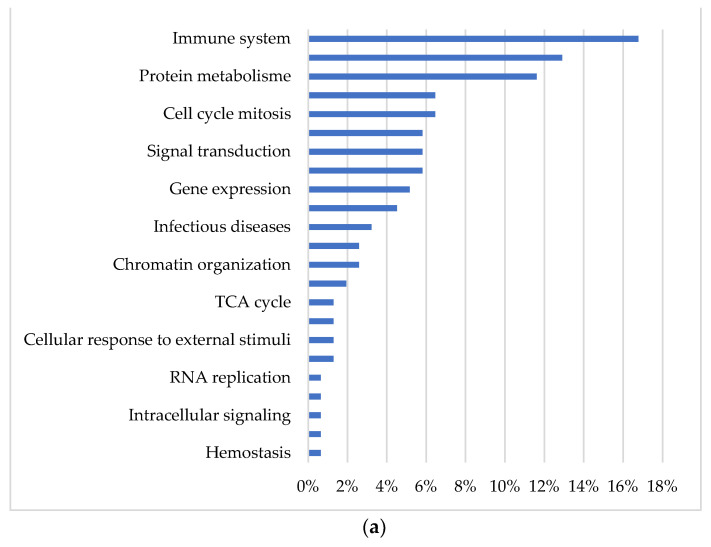

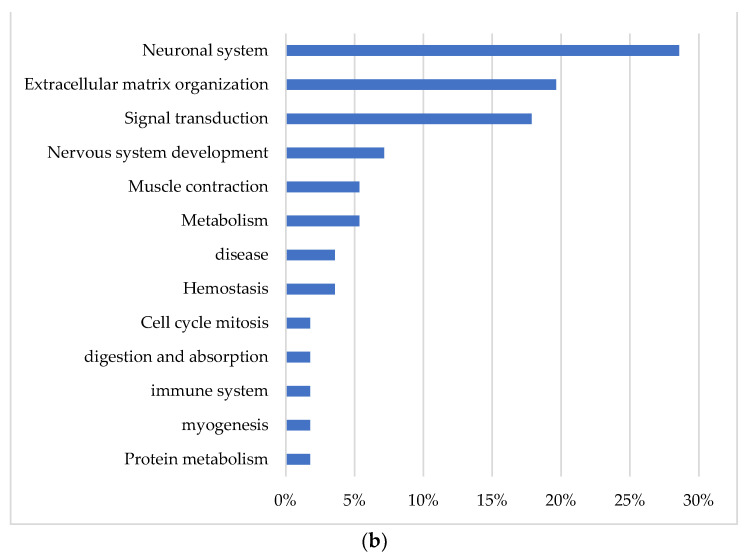
Pathways classified according to biological process according to their GO classification (**a**) Down-regulated pathways in the XLA group in relation to the PC group; (**b**) Up-regulated pathways in the XLA group in relation to the PC group. PC = Positive control with medicinal zinc oxide, XLA = Extra low protein diet with non-marked amino acids.

Figure 3 displays the significantly down- and up-regulated pathways in the XLA pigs when compared to the PC pigs classified according to their biological process. The top three down-regulated biological processes were immune function, DNA repair, and protein metabolism, whereas the top three up-regulated processes were neuronal system, extracellular matrix organisation, and signal transduction.

### 3.4. Blood Analysis

Biochemical analysis of blood parameters is presented in Table 5; due to contamination by surroundings during blood withdrawal, only 17 blood samples could be analysed in the PC group. Albumin, protein, creatinine, blood urea nitrogen (BUN), and potassium were all reduced significantly in the XLA group when compared to the PC group (*p* < 0.05), whereas aspartate–aminotransferase were increased in the XLA group (*p* < 0.05). 

### 3.5. Organ Measurements

The absolute and relative organ weights are presented in Table 6 for day 10 and 24 post-weaning. Overall, there was an effect of time (day 10 and 24 post-weaning) on all absolute and relative organ weights (*p* < 0.05), except for the relative stomach weight, which was not affected by age of the pig (*p* > 0.05). The SB pigs had a relatively increased empty stomach weight at both day 10 and day 24 when compared to the SP pigs (day 10: SB = 0.87 vs. SP = 0.79; day 24: SB = 0.89 vs. SP = 0.80, *p* < 0.01). The small intestines were relatively smaller in the XLA pigs at day 10 and 24 post-weaning when compared the NC and SB pigs (*p* < 0.05).

## 4. Discussion

The PC group with medicinal ZnO had markedly fewer diarrhoea-treated pigs during the entire nursery period when compared to the NC group, which is not surprising as medicinal ZnO has been thoroughly proven to reduce post-weaning diarrhoea in pigs [3,6,23]. Taking into account that a high level of dietary protein increases the amount of undigested protein in the colon available for fermentation [5,24], the results from this study also confirms that a reduced CP level can be used as a dietary measure to decrease diarrhoea in diets without medicinal ZnO. The CP levels of 15.4 and 15.1% in the XLA diet reduced diarrhoea even further than medicinal ZnO could accomplish, suggesting that reduced dietary protein from weaning to about 15 kg (phase 1 + 2) post-weaning can be an efficient tool to control diarrhoea, not unlike ZnO.

Even though all diets were optimised and supplemented according to standard AA levels, the growth performance in the SP and SB groups did not equal that of the PC group. The SP pigs had a lower ADG when compared to the PC pigs, which was not the case in the trial by Lynegaard et al. [19]. The current trial ended at day 39 post-weaning and around 22 kg (Table 4), whereas in Lynegaard et al. [19], the Phase 3 diets were allocated from 15 kg to approximately 30 kg (~day 48) and consequently, the pigs had more time in the high CP diet, which may explain the insignificant difference in growth performance between SP and PC in their trial. Previous results have also demonstrated a reduced ADG in pigs allocated less than 19% CP from weaning to 15 kg [6,24]. Moreover, previous results have suggested that substitution of soybean meal with soy protein concentrate could result in an improved nutrient utilisation and increased weight gain [7,12], as soy protein concentrate has fewer antinutritional factors and therefore contains more digestible CP [7,10]. However, this was not observed in the current study, as no differences were detected in diarrhoea-related antibiotic treatments or ADG between the SP and SB groups during the entire nursing period. 

The XLA group, receiving about 15% CP in both Phase 1 and 2 diets, demonstrated a lower ADG than the two control groups, PC and NC, as well as the SP and SB groups in the overall nursery period. This confirms the results of Lynegaard et al. [19], where the XLA group also displayed a reduced feed intake when compared to the PC and NC groups. In contrast, previous studies have demonstrated that inclusion of essential AAs (Lys, Met, Cys, Thr, Trp, Phe, Val, Ile, Leu, and His) in a l6.8% CP diet kept ADG at the same level as the control groups [13]. Results from the current trial suggest that the XLA diet were insufficient in covering the CP and essential AA requirements and we propose that the availability of nitrogen was not adequate to cover synthesising the non-essential AAs. Furthermore, the transcriptomic results demonstrated that several pathways involved with especially protein and AA metabolism were diminished in the XLA group, suggesting that the reduced growth performance may be a result of protein being prioritised for more essential biological processes, or that AA supply was insufficient. It can further be speculated whether the reduced growth performance of the XLA group may be caused by a lower feed intake, as seen in Lynegaard et al. [19].

The XLA group had down-regulation of numerous gene pathways related to protein metabolism, as well as down-regulation of several genes (e.g., *AICDA*, *STK17A*, *BATF*) and pathways involved with the immune response when compared to the PC group. The down-regulation of protein metabolism-related pathways agrees with the notion of an amino acid deficiency induced by the dietary treatment. Interestingly, up-regulated pathways were related to processes such as extracellular matrix remodelling, which suggests that diverse biochemical pathways are altered by the dietary treatments, and further research should focus on the functional implications of this on health and performance. The suppression of immune-related pathways agrees with the reduced frequency of diarrhoea observed in the XLA group, indicating that a 15.4% CP level stimulated gut health similar to medicinal ZnO. Previous results on transcriptome and qPCR analysis revealed that ZnO in the diet of newly weaned pigs challenged with Enterotoxigenic E. coli (ETEC) had a significant effect on the innate immune system and zinc absorption [25]. On the other hand, the suppression of genes involved with immune response could also be a disadvantage for the pigs in the current study, but the reduced diarrhoea in the XLA group indicated that they could mount an immune response even without the inclusion of ZnO. Similarly, Sargeant et al. [25] suggested that the most significant effect of ZnO treatment was on the immune response genes. Perhaps, the suppressed genes involved with the immune response in combination with a low diarrhoea occurrence in the XLA pigs suggest that the small proportion of diarrhoea cases were caused by undigested proteins and not pathogens, which should have resulted in an immune response. At the same time, it may not be surprising that immune genes were down-regulated, as protein is part of the immune process [26]. It can also be speculated as to whether the suppressed genes involved with immune response in the XLA pigs was caused by an undersupply of protein, as also protein metabolism was suppressed.

It is documented that AAs regulate several metabolic pathways which are crucial for health, maintenance, and growth in pigs [27]. The XLA diet was supplemented with essential AAs to reduce the negative impact of the low CP level on growth performance, which was not a success in the current trial. However, previous results indicate that a 14% CP diet supplemented with crystalline AA may not provide sufficient AA levels to maintain growth performance [28]. The absorption of AAs require many different transporter systems [29], and it has been reported that some genes involved with the control of growth and AA metabolism are regulated by AA availability [27,30,31]. The transcriptomic results from the current study show that several genes involved with both protein and AA metabolism were down-regulated in the XLA group. This corroborates previous results, where a 14% CP and AA-supplemented diet resulted in reduced expression of AA transporter genes in the jejunum [28], suggesting that a low CP level may reduce the capacity of AA transport in the small intestine. Furthermore, it can be speculated whether dietary CP levels can only be reduced within an appropriate range, and that any further reductions may dramatically alter AA utilisation in the small intestine of the pig [32]. 

Not surprisingly, BUN was lower in XLA pigs 24 days post-weaning, as they received a reduced level of CP in both Phase 1 and 2 when compared to the PC pigs, which is consistent with results from Larsen et al. [33]. The concentration of BUN is an indicator of protein utilisation [34,35], as it is correlated with urinary nitrogen excretion, and can therefore be used to predict nitrogen excretion and the efficiency of nitrogen utilisation in pigs [36]. In previous studies, a decrease in BUN was reported when dietary CP levels were reduced post-weaning [37,38,39]. It has further been suggested that BUN concentrations decrease with reduced CP levels due to optimal utilisation of AA and less nitrogen intake [39]. The decreased concentration of BUN as a result of the low CP level in the XLA diet of the current study indicates that nitrogen excretion was reduced, which is consistent with results by Yu et al. [40]. As the growth performance was not equal between the XLA and PC groups, it can again be speculated whether nitrogen was in undersupply, and that the low BUN levels does not correspond with a higher utilization of nitrogen in the XLA pigs. 

Furthermore, XLA pigs had a reduced concentration of blood total protein, albumin, and creatinine in the current study, consistent with previous studies [41,42]. These results are not surprising, with albumin being the most important blood protein, as there is a positive correlation between blood protein and protein intake [42]. Additionally, blood protein is very sensitive to dietary influences, making the interpretation difficult [43]. Elevated levels of blood creatinine, as seen in the PC pigs of the current study, may indicate that the animals are going through greater muscular development [43], which corresponds with the increased growth of the PC pigs when compared with the XLA pigs. On the other hand, lower creatinine levels may also express an overall better AA status [44]. However, as creatinine is synthesised in the body from glycine, arginine, and methionine [45,46], the decreased creatinine levels taken together with the low growth performance of XLA pigs, suggest that they were deficient in essential AAs.

The organ measurements demonstrate a reduced relative empty small intestinal weight in the XLA group at both day 10 and 24 post-weaning, which is not only a result of their lower bodyweight at the sampling time. The reduced protein levels may therefore not only reduce growth performance but may also inhibit the maturation and growth of essential digestive organs such as the small intestine. This in turn decreases the absorptive area and capacity of the small intestine and could result in even further reduced growth in the XLA group. 

## 5. Conclusions

In conclusion, the data suggest that the XLA diet may have been insufficient in protein and AAs but could potentially make the post-weaning period easier for pigs by decreasing diarrhoea and thereby antibiotics treatments. Furthermore, the results revealed only minor differences in diarrhoea and growth performance between the SP and SB groups, indicating that both protein sources can equally well be allocated in 17.6% CP diet post-weaning. The decreased activity of several genes involved with nutrient metabolism and immune response in the XLA group when compared to the PC group, together with the decreased diarrhoea, growth performance, and blood protein levels, suggest that the piglets allocated a 15.4% CP diet post-weaning may have a redistribution of available protein towards essential processes, thereby compromising growth. 

## Figures and Tables

**Table 1:** Analysed nutritional contents of the five experimental diets in the three feeding phases for weaned pigs.

Dietary Treatment ^1^	Phase 1					Phase 2				Phase 3	
	PC	NC	SP	SB	XLA	PC NC	SP	SB	XLA	PC NC	SP SB XLA
Chemical composition											
CP^2^, g/kg	187.9	188.4	176.0	175.9	155.3	187.2	176.0	176.1	151.4	190.6	190.0
Calcium, g/kg	7.5	8.0	7.3	7.1	7.3	8.0	6.9	7.1	7.8	8.8	8.6
Phosphorous, g/kg	6.5	6.0	6.1	5.0	6.1	6.1	6.1	6.1	6.2	5.4	5.4
Zinc, mg/kg	2740	140	138	166	70	139	138	132	130	144	135
Copper, mg/kg	150	125	125	122	38	93	96	88	71	74	65
Total amino acids, g/kg											
Lys	12.9	13.6	13.2	13.2	13.0	13.3	13.5	13.1	13.0	13.2	12.8
Met	4.2	4.3	4.2	4.3	4.5	4.0	4.2	4.0	4.4	4.0	3.8
Met + Cys	7.1	7.2	6.9	7.1	6.9	7.0	7.1	6.8	6.8	7.0	6.9
Thr	8.3	8.5	8.3	8.5	8.2	8.4	8.5	8.4	7.9	8.0	8.1
Val	9.4	9.4	8.7	8.9	8.6	9.3	8.7	8.7	8.4	9.0	9.1
His	4.1	4.2	3.8	3.8	3.7	4.3	3.9	4.0	3.6	4.3	4.4
Ile	7.3	7.4	6.7	6.8	6.1	7.3	6.7	6.6	6.2	6.9	7.0
Leu	13.7	13.9	12.6	12.8	12.0	13.6	12.6	12.3	11.9	12.7	12.8
Phe	9.1	9.2	8.4	8.4	7.3	9.1	8.4	8.3	7.3	6.6	8.7
Digestible amino acids ^2^, g/kg											
SID CP	164.3	165.1	154.1	154.0	135.9	164.4	153.9	152.8	155.8	166.7	166.4
SID Lys	11.6	12.3	12.0	12.0	12.0	12.1	12.4	12.2	12.1	12.0	11.6
SID Met	3.9	3.9	3.9	4.0	4.3	3.7	4.0	3.7	4.1	3.7	3.6
SID Met + Cys	6.2	6.3	6.1	6.3	6.2	6.2	6.2	6.0	6.1	6.2	6.1
SID Thr	7.3	7.4	7.4	7.5	7.4	7.4	7.5	7.3	7.1	7.0	7.1
SID Val	8.1	8.1	7.5	7.6	7.5	8.1	7.5	7.5	7.4	7.8	7.9
SID His	3.6	3.7	3.3	3.3	3.2	3.7	3.4	3.5	3.2	3.7	3.8
SID Ile	6.4	6.5	5.8	5.8	5.4	6.3	5.9	5.7	5.5	6.1	6.1
SID Leu	12.2	12.3	11.2	11.3	10.7	11.9	11.0	10.8	10.5	11.2	11.3
SID CP	164.3	165.1	154.1	154.0	135.9	164.4	153.9	152.8	155.8	166.7	166.4

^1^ PC = Positive control with medicinal zinc oxide; NC = Negative control without medicinal zinc oxide, SP = Soy protein concentrate, SB = Soybean meal, XLA = X-low protein + amino acids. ^2^ CP = Crude protein; SID = Standardised ileal digestible: The content of SID CP and amino acids were calculated based on analyzed total values of the six dietary treatments and on SID digestibility coefficients of the feed ingredients from Danish Agro (Sjölund, Denmark).

**Table 2:** Composition of the five experimental diets for weaned pigs in a three-phase feeding programme.

Dietary Treatment ^1^ Ingredient (%)	Phase 1					Phase 2				Phase 3	
	PC	NC	SP	SB	XLA	PC NC	SP	SB	XLA	PC NC	SP SB XLA
Wheat	46.6	47.7	53.0	51.7	59.4	52.1	59.1	54.9	65.4	49.8	49.7
Barley	20.0	20.0	20.0	20.0	20.0	20.0	20.0	20.0	20.0	20.0	20.0
Soybean meal	7.0	7.0	0.5	7.0	0.5	14.0	6.0	14.0	2.2	21.0	22.5
Soy protein concentrate ^2^	6.5	6.4	7.5	2.2	0	2.9	2.6	0.85	0	2.1	0.5
Potato protein conc.	4.0	4.0	4.0	4.0	4.0	3.0	3.0	2.0	3.0	0	0
Fishmeal	2.0	2.0	2.0	2.0	2.0	0	2.0	0	0.5	0	0
Whey powder	6.0	6.0	6.0	6.0	6.0	0	0	0	0	0	0
Fatty acid distillate	2.4	2.1	1.7	1.9	1.2	2.7	1.9	2.5	1.5	1.9	2.0
Monocalcium phosphate	1.4	1.2	1.3	1.3	1.5	0.2	0.1	0.2	0.1	0.9	0.9
Limestone	0	0	0	0	0	0	0	0	0	1.5	1.5
Sodium chloride	0.6	0.6	0.6	0.6	0.6	1.3	1.2	1.3	1.5	0.5	0.5
Sodium bicarbonate	0.1	0.1	0.1	0.1	0.1	0.6	0.6	0.6	0.6	0.1	0.1
Lysine sulphate 70%	0.69	0.69	0.90	0.86	1.25	0.1	0.1	0.1	0.1	0.72	0.81
Methionine 98%	0.11	0.11	0.15	0.15	0.23	0.76	0.93	0.93	1.35	0.14	0.16
Threonine 98%	0.13	0.13	0.20	0.20	0.33	0.13	0.15	0.17	0.24	0.17	0.21
Tryptophan 99%	0.05	0.05	0.07	0.07	0.12	0.16	0.22	0.23	0.36	0.03	0.04
Valine 96.5%	0.03	0.03	0.05	0.05	0.20	0.05	0.07	0.06	0.12	0.06	0.09
Mineral-vitamin premix ^3^	0.40	0.40	0.40	0.40	0.40	0.05	0.07	0.08	0.23	0.40	0.40
Phytase ^4^	0.03	0.03	0.03	0.03	0.03	0.40	0.40	0.40	0.40	0.03	0.03
Benzoic acid	0.50	0.50	0.50	0.50	0.50	0.03	0.03	0.03	0.03	0.50	0.50
Calcium formate	1.00	1.00	1.00	1.00	1.00	0.50	0.50	0.50	0.50	0	0
Microgrits Green ^5^	0.05	0	0	0	0	1.00	1.00	1.00	1.00	0.05	0
Microgrits Blue ^5^	0	0	0.05	0	0	0.05	0	0	0	0	0.05
Zinc oxide	0.30	0	0	0	0	0	0.05	0	0	0	0
Isoleucine 98.5%	0	0	0	0	0.12	0	0	0	0.14	0	0
Leucine 98.5%	0	0	0	0	0.22	0	0	0	0.26	0	0
Histidine 98.5%	0	0	0	0	0.08	0	0	0	0.09	0	0
Phenylalanine 98.5%	0	0	0	0	0.08	0	0	0	0.1	0	0
Tyrosine 98.5%	0	0	0	0	0.15	0	0	0	0.17	0	0

^1^ PC = Positive control with medicinal zinc oxide; NC = Negative control without medicinal zinc oxide, SP = Soy protein concentrate, SB = Soybean meal, XLA = X-low protein + amino acids. ^2^ Vilosoy from Vilomix (Sjølund, Denmark). ^3^ DA Vit weaning mix from Danish Agro (Sjølund, Denmark), DA = Danish Agro. ^4^ DSM Ronozyme, phytase enzyme from DSM (Brøndby, Denmark). ^5^ Microgrits were added, to ensure that the correct feed was delivered from the correct silos.

**Table 3:** Percentage of cumulative antibiotics (AB)-treated pigs and the number of pigs removed from the trial in each of the dietary groups at days 10, 24, and 39 post-weaning.

	Dietary Group ^1^					*p*-Value
	PC	NC	SP	SB	XLA	
Pigs (n)	583	1165	582	584	584	
AB treated pigs (%)						
Day 10	1.9 ^a^	12.1 ^b^	11.2 ^b^	9.6 ^b^	2.9 ^a^	<0.001
Day 24	32.7 ^a^	50.8 ^b^	40.5 ^c^	44.8 ^c^	11.8 ^d^	<0.001
Day 39	43.5 ^a^	63.9 ^b^	53.4 ^c^	52.1 ^c^	24.4 ^d^	<0.001
Removed pigs						
Day 10	2	3	2	0	0	ns
Day 24	14	58	12	10	14	ns
Day 39	25	102	19	26	22	ns

^1^ PC = Positive control with medicinal zinc oxide, NC = Negative control, SP = Low protein diet with soy protein concentrate as primary protein source, SB = Low protein diet with soybean meal as primary protein source, XLA = Extra low protein diet with non-marked amino acids. ^a,b,c,d^ Values within a row with different superscripts differ significantly at *p* < 0.05.

**Table 4:** Mean bodyweight and daily weight gain in the dietary groups of the antibiotics (AB)-treated and untreated pigs.

AB	Dietary Treatment ^1^										SEM	*p*-Value	
	PC	NC	SP	SB	XLA	PC	NC	SP	SB	XLA			
	Untreated					Treated						Diet	AB ^2^
Pigs (n)													
d 10	569	1021	515	528	566	11	141	65	56	17	-		-
d 24	369	534	327	305	484	179	552	223	248	65	-		
d 39	293	369	244	249	394	226	652	652	271	127	-		
Bodyweight (kg)													
d 10	8.35 ^a^	8.23 ^a,b^	8.21 ^a,b^	8.24 ^a,b^	8.09 ^b^	8.39 ^a^	8.27 ^a,b^	8.26 ^a,b^	8.28 ^a,b^	8.13 ^b^	0.18	0.009	0.63
d 24	13.18 ^a^	12.48 ^b^	12.18 ^b^	12.62 ^b^	11.40 ^d^	13.61 ^a^	12.91 ^b^	12.61 ^b^	13.06 ^b^	11.84 ^d^	0.28	<0.001	<0.001
d 39	22.68 ^a^	21.94 ^a,b^	21.07 ^b^	21.67 ^a,b^	19.38 ^c^	23.10 ^a^	22.37 ^a,b^	21.49 ^b^	22.10 ^a,b^	19.80 ^c^	0.55	<0.001	0.14
ADG ^2^ (g/d)													
d 1–10	138.48 ^a^	130.62 ^a^	129.01 ^a^	131.23 ^a^	115.99 ^b^	118.36 ^a^	110.50 ^a^	108.88 ^a^	111.11 ^a^	95.87 ^b^	8.75	<0.001	<0.001
d 10–24	346.20 ^a^	303.73 ^b^	286.19 ^c^	314.75 ^b^	234.31 ^d^	364.56 ^a^	322.09 ^b^	304.55 ^c^	333.10 ^b^	252.67 ^d^	8.97	<0.001	<0.001
d 24–39	768.35 ^a^	759.12 ^a^	707.79 ^a,b^	729.41 ^a,b^	656.93 ^b^	785.34 ^a^	776.11 ^a^	724.79 ^a,b^	746.39 ^a,b^	673.92 ^b^	107.8	<0.001	0.36
d 1–39	420.85 ^a^	401.14 ^a,b^	377.53 ^b^	393.88 ^a,b^	332.73 ^c^	427.80 ^a^	408.09 ^a,b^	384.48 ^b^	400.83 ^a,b^	339.68 ^c^	16.45	<0.001	0.34

^1^ PC = Positive control with medicinal zinc oxide, NC = Negative control, SP = Low protein diet with soy protein concentrate as primary protein source, SB = Low protein diet with soybean meal as primary protein source, XLA = Extra low protein diet with non-marked amino acids. ^2^ AB = antibiotics, ADG = average daily gain; ^a,b,c,d^ Values within a row (untreated or treated) with different superscripts differ significantly at *p* < 0.05.

**Table 5:** Biochemical blood parameters analysis of the PC and XLA pigs at 24 days post-weaning.

	Dietary Group ^1^		SEM	*p*-Value
	PC	XLA		
Pigs (n)	17	20		
Albumin (g/L)	30.5	26.5	1.09	0.014
Protein (g/L)	58.3	50.4	2.13	0.013
Alkaline phosphatase (U/L)	565	559	38.7	0.913
Alanine–aminotransferase (U/L)	60.4	66.9	5.14	0.377
Bilirubin (umol/L)	0.06	0.19	0.13	0.478
Cholesterol (mmol/L)	2.20	2.09	0.15	0.596
Creatinine (umol/L)	65.8	57.0	2.96	0.043
Iron (mmol/L)	30.1	31.4	3.04	0.777
Phosphate (mmol/L)	3.31	3.25	0.12	0.725
Aspartate–aminotransferase (U/L)	72.4	178.2	39.0	0.064
Blood urea nitrogen (mmol/L)	1.85	0.89	0.22	0.005
Gamma–glutamyl transferase (U/L)	30.8	28.5	2.60	0.528
Calcium (mmol/L)	3.01	2.75	0.10	0.069
Magnesium (mmol/L)	1.04	0.91	0.06	0.162
Sodium (mmol/L)	161	148	4.83	0.079
Potassium (mmol/L)	7.93	7.00	0.31	0.046

^1^ PC = Positive control group with medicinal zinc oxide; XLA = Extra low protein diet with non-marked amino acids.

**Table 6:** Effect of dietary treatment on organ weights at day 10 and 24 post-weaning in dietary groups.

Column Head	Dietary Treatment ^1^										SEM	*p*-Value	
	PC	NC	SP	SB	XLA	PC	NC	SP	SB	XLA			
	Day 10 Post-Weaning					Day 24 Post-Weaning						Diet	Age
n	20	20	20	20	20	18	20	19	17	20			
Bodyweight (kg)	8.93 ^a^	8.32 ^a,b^	8.99 ^a^	8.30 ^a,b^	7.68 ^b^	13.61	12.98	13.66	12.97	12.36	0.419	0.025	<0.001
Absolute organ weight													
Full stomach (g)	362.6	337.3	344.2	384.7	313.8	547.8	522.6	529.4	569.9	499.0	41.06	0.305	0.002
Empty stomach (g)	75.5 ^a^	71.7 ^a,b^	68.9 ^a,b^	73.8 ^a^	63.7 ^b^	115.1 ^a^	111.3 ^a,b^	108.5 ^a,b^	113.4 ^a^	103.2 ^b^	4.73	0.019	<0.001
Full small intestine (g)	664.9 ^a^	655.6 ^a^	676.2 ^a^	646.5 ^a^	495.8 ^b^	1166.2 ^a^	1157.0 ^a^	1177.6 ^a^	1147.9 ^a^	997.2 ^b^	47.60	<0.001	<0.001
Empty small intestine (g)	408.7 ^a^	396.9 ^a^	417.0 ^a^	395.6 ^a^	320.0 ^b^	730.7 ^a^	719.6 ^a^	739.6 ^a^	718.2 ^a^	642.6 ^b^	27.88	0.003	<0.001
Full colon (g)	379.2 ^a^	347.4 ^a^	334.1	343.9 ^a^	264.1 ^b^	651.6 ^a^	619.8 ^a^	606.5	616.4 ^a^	536.4 ^b^	26.36	0.002	<0.001
Empty colon (g)	148.6 ^a^	140.3 ^a,b^	137.7 ^a,b^	138.9 ^a,b^	119.5 ^b^	248.6 ^a^	240.3 ^a,b^	237.7 ^a,b^	238.9 ^a,b^	219.5 ^b^	7.61	0.025	<0.001
Small intestine length (m)	9.74	9.63	9.96	9.74	9.38	11.29	11.19	11.51	11.29	10.93	0.436	0.247	0.015
Relative organ weight (%)													
Full stomach	4.03	4.01	3.93	4.53	4.06	3.98	3.95	3.89	4.48	4.00	0.260	0.232	0.824
Empty stomach	0.85 ^a,b^	0.85 ^a,b^	0.79 ^a^	0.87^b^	0.83 ^a,b^	0.86 ^a,b^	0.86 ^a,b^	0.80 ^a^	0.89 ^b^	0.84 ^a,b^	0.026	0.016	0.662
Full small intestine	7.36 ^a,b^	7.75 ^a^	7.50 ^a^	7.67 ^a^	6.76 ^b^	8.59 ^a,b^	8.98 ^a^	8.73 ^a^	8.89 ^a^	7.99 ^b^	0.262	0.005	<0.001
Empty small intestine	4.48 ^a,b^	4.73 ^a^	4.64 ^a,b^	4.70 ^a^	4.31 ^b^	5.34 ^a,b^	5.59 ^a^	5.50 ^a,b^	5.57 ^a^	5.17 ^b^	0.155	0.029	<0.001
Full colon	4.13	4.13	3.84	4.16	3.56	4.83	4.83	4.54	4.86 ^a^	4.25 ^b^	0.192	0.023	0.002
Empty colon	1.64	1.68	1.57	1.67	1.58	1.83	1.88	1.77	1.87	1.77	0.055	0.166	0.004
Small intestine length	1.13	1.16	1.14	1.18	1.19	0.83	0.87	0.85	0.89	0.90	0.031	0.193	<0.001

^1^ PC = Positive control with medicinal zinc oxide, NC = Negative control, SP = Low protein diet with soy protein concentrate as primary protein source, SB = Low protein diet with soybean meal as primary protein source, XLA = Extra-low protein diet with non-marked amino acids, SI = Small intestine. ^a,b^ Values within a row with different superscripts differ significantly at *p* < 0.05.

## Data Availability

Data are available upon request and are not publicly available due to practicalities.

## Supplementary Materials

The following supporting information can be downloaded at: https://www.mdpi.com/article/10.3390/ani12080989/s1, Table S1: GO enrichment analysis, up-regulated pathways in the XLA group compared to the PC group. Table S2. GO enrichment analysis, down-regulated pathways in the XLA group compared to the PC group.
