# Supplementary material for: Reduction in Diarrhoea and Modulation of Intestinal Gene Expression in Pigs Allocated a Low Protein Diet without Medicinal Zinc Oxide Post-Weaning
Source: Animals (Basel). 2022 Apr 11;12(8):989. doi: 10.3390/ani12080989 (PMC9027983; doi:10.3390/ani12080989)
Supplement: Supplementary file 1 [file animals-12-00989-s001.zip › animals-1643839-supplementary.pdf]

Reduction in diarrhoea and modulation of intestinal gene expression in pigs allocated a low protein diet without medicinal zinc oxide post-weaning

J.C. Lynegaard[1], N.J. Kjeldsen[2], C.F. Hansen[2], A.R. Williams[1], J.P. Nielsen[1] and C. Amdi[1]

[1]Department of Veterinary and Animal Sciences, Faculty of Health and Medical Sciences, University of Copenhagen, Denmark
[2]Pig Research Centre, Danish Agriculture and Food Council, Axeltorv 3, DK-1609 Copenhagen V, Denmark

Supplementary Materials

Table S1. GO enrichment analysis, up-regulated pathways in the XLA group compared to the PC group

NAME
NABA_CORE_MATRISOME
REACTOME_ECM_PROTEOGLYCANS
REACTOME_EXTRACELLULAR_MATRIX_ORGANIZATION
NABA_ECM_GLYCOPROTEINS
REACTOME_NON_INTEGRIN_MEMBRANE_ECM_INTERACTIONS
KEGG_ECM_RECEPTOR_INTERACTION
REACTOME_NEURONAL_SYSTEM
NABA_COLLAGENS
REACTOME_COLLAGEN_CHAIN_TRIMERIZATION
NABA_BASEMENT_MEMBRANES
PID_INTEGRIN1_PATHWAY
REACTOME_NCAM1_INTERACTIONS
REACTOME_INTEGRIN_CELL_SURFACE_INTERACTIONS
REACTOME_COLLAGEN_FORMATION
REACTOME_ASSEMBLY_OF_COLLAGEN_FIBRILS_AND_OTHER_MULTIMERIC_STRUCTURES
REACTOME_COLLAGEN_BIOSYNTHESIS_AND_MODIFYING_ENZYMES
REACTOME_TRANSMISSION_ACROSS_CHEMICAL_SYNAPSES
REACTOME_DEGRADATION_OF_THE_EXTRACELLULAR_MATRIX
NABA_PROTEOGLYCANS
REACTOME_MUSCLE_CONTRACTION
KEGG_FOCAL_ADHESION
REACTOME_MET_ACTIVATES_PTK2_SIGNALING
REACTOME_GPCR_LIGAND_BINDING
REACTOME_PROTEIN_PROTEIN_INTERACTIONS_AT_SYNAPSES
PID_UPA_UPAR_PATHWAY
REACTOME_AMINE_LIGAND_BINDING_RECEPTORS
NABA_ECM_REGULATORS
REACTOME_ACTIVATION_OF_NMDA_RECEPTORS_AND_POSTSYNAPTIC_EVENTS
REACTOME_CGMP_EFFECTS

REACTOME_INWARDLY_RECTIFYING_K_CHANNELS

REACTOME_CROSSLINKING_OF_COLLAGEN_FIBRILS

REACTOME_PLATELET_HOMEOSTASIS

REACTOME_UNBLOCKING_OF_NMDA_RECEPTORS_GLUTAMATE_BINDING_AND_ACTIVATION

REACTOME_CLASS_B_2_SECRETIN_FAMILY_RECEPTORS

REACTOME_ION_HOMEOSTASIS

REACTOME_GABA_RECEPTOR_ACTIVATION

REACTOME_ANTIVIRAL_MECHANISM_BY_IFN_STIMULATED_GENES

REACTOME_ACETYLCHOLINE_NEUROTRANSMITTER_RELEASE_CYCLE

REACTOME_EGR2_AND_SOX10_MEDIATED_INITIATION_OF_SCHWANN_CELL_MYELINATION

PID_WNT_SIGNALING_PATHWAY

REACTOME_L1CAM_INTERACTIONS

KEGG_HYPERTROPHIC_CARDIOMYOPATHY_HCM

REACTOME_ACTIVATION_OF_KAINATE_RECEPTORS_UPON_GLUTAMATE_BINDING

REACTOME_DISEASES_ASSOCIATED_WITH_O_GLYCOSYLATION_OF_PROTEINS

REACTOME_MYOGENESIS

REACTOME_ORGANELLE_BIOGENESIS_AND_MAINTENANCE

REACTOME_DISEASES_OF_GLYCOSYLATION

REACTOME_CA_DEPENDENT_EVENTS

REACTOME_RECEPTOR_TYPE_TYROSINE_PROTEIN_PHOSPHATASES

REACTOME_SMOOTH_MUSCLE_CONTRACTION

REACTOME_CILIUM_ASSEMBLY

REACTOME_LONG_TERM_POTENTIATION

PID_TELOMERASE_PATHWAY

REACTOME_O_LINKED_GLYCOSYLATION

BIOCARTA_CHREBP_PATHWAY

REACTOME_ADRENALINE_NORADRENALINE_INHIBITS_INSULIN_SECRETION

REACTOME_VOLTAGE_GATED_POTASSIUM_CHANNELS

KEGG_DILATED_CARDIOMYOPATHY

REACTOME_REGULATION_OF_PLK1_ACTIVITY_AT_G2_M_TRANSITION

REACTOME_DAG_AND_IP3_SIGNALING

REACTOME_G_PROTEIN_MEDIATED_EVENTS

REACTOME_NEGATIVE_REGULATION_OF_NMDA_RECEPTOR_MEDIATED_NEURONAL_TRANSMISSION

REACTOME_DIGESTION_AND_ABSORPTION

REACTOME_ASSEMBLY_AND_CELL_SURFACE_PRESENTATION_OF_NMDA_RECEPTORS

KEGG_BASAL_CELL_CARCINOMA

REACTOME_CREB1_PHOSPHORYLATION_THROUGH_NMDA_RECEPTOR_MEDIATED_ACTIVATION_OF_RAS_SIGNALING

REACTOME_ANCHORING_FIBRIL_FORMATION

REACTOME_GLUCAGON_SIGNALING_IN_METABOLIC_REGULATION

REACTOME_FATTY_ACYL_COA_BIOSYNTHESIS

REACTOME_NEUREXINS_AND_NEUROLIGINS

REACTOME_OPIOID_SIGNALLING

REACTOME_VISUAL_PHOTOTRANSDUCTION

REACTOME_NETRIN_1_SIGNALING

REACTOME_GLUTAMATE_NEUROTRANSMITTER_RELEASE_CYCLE

Table S2. GO enrichment analysis, down-regulated pathways in the XLA group compared to the PC group

NAME
REACTOME_SELENOAMINO_ACID_METABOLISM
REACTOME_METABOLISM_OF_AMINO_ACIDS_AND_DERIVATIVES
REACTOME_M_PHASE
REACTOME_CELL_CYCLE_CHECKPOINTS
REACTOME_MITOTIC_METAPHASE_AND_ANAPHASE
REACTOME_SEPARATION_OF_SISTER_CHROMATIDS
REACTOME_CELL_CYCLE_MITOTIC
REACTOME_RESPONSE_OF_EIF2AK4_GCN2_TO_AMINO_ACID_DEFICIENCY
REACTOME_CELLULAR_RESPONSES_TO_EXTERNAL_STIMULI
REACTOME_CHROMATIN_MODIFYING_ENZYMES
REACTOME_INFLUENZA_INFECTION
REACTOME_DNA_REPAIR
REACTOME_HIV_INFECTION
REACTOME_REGULATION_OF_EXPRESSION_OF_SLITS_AND_ROBOS
REACTOME_SIGNALING_BY_ROBO_RECEPTORS
REACTOME_EUKARYOTIC_TRANSLATION_ELONGATION
REACTOME_SRP_DEPENDENT_COTRANSLATIONAL_PROTEIN_TARGETING_TO_MEMBRANE
REACTOME_EUKARYOTIC_TRANSLATION_INITIATION
REACTOME_MITOCHONDRIAL_TRANSLATION
REACTOME_ACTIVATION_OF_THE_MRNA_UPON_BINDING_OF_THE_CAP_BINDING_COMPLEX_AND_EIFS_AND_SUBSEQUENT_BINDING_TO_43S
REACTOME_TRANSLATION
REACTOME_NONSENSE_MEDIATED_DECAY_NMD
REACTOME_PROCESSING_OF_CAPPED_INTRON_CONTAINING_PRE_MRNA
REACTOME_MRNA_SPLICING
REACTOME_TRANSCRIPTIONAL_REGULATION_BY_TP53
REACTOME_TP53_REGULATES_TRANSCRIPTION_OF_DNA_REPAIR_GENES
KEGG_RIBOSOME
KEGG_SPLICEOSOME
KEGG_OXIDATIVE_PHOSPHORYLATION
REACTOME_DNA_DOUBLE_STRAND_BREAK_REPAIR
REACTOME_SIGNALING_BY_THE_B_CELL_RECEPTOR_BCR
REACTOME_FORMATION_OF_RNA_POL_II_ELONGATION_COMPLEX
REACTOME_ASPARAGINE_N_LINKED_GLYCOSYLATION
REACTOME_TRANSCRIPTION_OF_THE_HIV_GENOME
REACTOME_REGULATION_OF_TP53_ACTIVITY
REACTOME_DEGRADATION_OF_BETA_CATENIN_BY_THE_DESTRUCTION_COMPLEX
REACTOME_APC_C_MEDIATED_DEGRADATION_OF_CELL_CYCLE_PROTEINS
REACTOME_HIV_TRANSCRIPTION_ELONGATION
KEGG_PRIMARY_IMMUNODEFICIENCY

REACTOME_PROCESSING_OF_DNA_DOUBLE_STRAND_BREAK_ENDS
REACTOME_RNA_POLYMERASE_II_TRANSCRIBES_SNRNA_GENES
REACTOME_REGULATION_OF_RUNX3_EXPRESSION_AND_ACTIVITY
REACTOME_SUMOYLATION
REACTOME_UNFOLDED_PROTEIN_RESPONSE_UPR
KEGG_UBIQUITIN_MEDIATED_PROTEOLYSIS
REACTOME_RNA_POLYMERASE_II_TRANSCRIPTION_TERMINATION
REACTOME_GLOBAL_GENOME_NUCLEOTIDE_EXCISION_REPAIR_GG_NER
REACTOME_DNA_REPLICATION_PRE_INITIATION
REACTOME_FCERI_MEDIATED_CA_2_MOBILIZATION
REACTOME_RESPIRATORY_ELECTRON_TRANSPORT
REACTOME_DUAL_INCISION_IN_TC_NER
REACTOME_NEGATIVE_REGULATION_OF_NOTCH4_SIGNALING
REACTOME_HOST_INTERACTIONS_OF_HIV_FACTORS
REACTOME_UCH_PROTEINASES
REACTOME_PROTEIN_UBIQUITINATION
REACTOME_PTEN_REGULATION
KEGG_T_CELL_RECEPTOR_SIGNALING_PATHWAY
REACTOME_SWITCHING_OF_ORIGINS_TO_A_POST_REPLICATIVE_STATE
REACTOME_FCERI_MEDIATED_NF_KB_ACTIVATION
REACTOME_ANTIGEN_PROCESSING_CROSS_PRESENTATION
REACTOME_COMPLEX_I_BIOGENESIS
REACTOME_CLEC7A_DECTIN_1_SIGNALING
REACTOME_DNA_STRAND_ELONGATION
REACTOME_DEGRADATION_OF_DVL
PID_NFAT_TFPATHWAY
REACTOME_TRANSPORT_TO_THE_GOLGI_AND_SUBSEQUENT_MODIFICATION
REACTOME_ER_TO_GOLGI_ANTEROGRADE_TRANSPORT
REACTOME_INTERLEUKIN_3_INTERLEUKIN_5_AND_GM_CSF_SIGNALING
REACTOME_APC_C_CDH1_MEDIATED_DEGRADATION_OF_CDC20_AND_OTHER_APC_C_CDH1_TARGETED_PROTEINS_IN_LATE_MITOSIS_EARLY_G1
REACTOME_STABILIZATION_OF_P53
REACTOME_DEGRADATION_OF_GLI1_BY_THE_PROTEASOME
REACTOME_HDR_THROUGH_HOMOLOGOUS_RECOMBINATION_HRR
PID_ATR_PATHWAY
REACTOME_FORMATION_OF_TC_NER_PRE_INCISION_COMPLEX
KEGG_B_CELL_RECEPTOR_SIGNALING_PATHWAY
REACTOME_C_TYPE_LECTIN_RECEPTORS_CLRS
REACTOME_HEDGEHOG_LIGAND_BIOGENESIS
REACTOME_FORMATION_OF_THE_EARLY_ELONGATION_COMPLEX
KEGG_NUCLEOTIDE_EXCISION_REPAIR
REACTOME_DEFECTIVE_CFTR_CAUSES_CYSTIC_FIBROSIS
REACTOME_BASE_EXCISION_REPAIR
REACTOME_TCR_SIGNALING

REACTOME_G1_S_DNA_DAMAGE_CHECKPOINTS
REACTOME_HIV_ELONGATION_ARREST_AND_RECOVERY
REACTOME_MITOTIC_G1_PHASE_AND_G1_S_TRANSITION
KEGG_NATURAL_KILLER_CELL_MEDIATED_CYTOTOXICITY
REACTOME_REGULATION_OF_TP53_ACTIVITY_THROUGH_PHOSPHORYLATION
REACTOME_CHROMOSOME_MAINTENANCE
REACTOME_NEGATIVE_EPIGENETIC_REGULATION_OF_RRNA_EXPRESSION
REACTOME_TRANSPORT_OF_MATURE_TRANSCRIPT_TO_CYTOPLASM
REACTOME_ASSEMBLY_OF_THE_PRE_REPLICATIVE_COMPLEX
KEGG_BASE_EXCISION_REPAIR
REACTOME_THE_ROLE_OF_GTSE1_IN_G2_M_PROGRESSION_AFTER_G2_CHECKPOINT
REACTOME_G2_M_DNA_DAMAGE_CHECKPOINT
REACTOME_TNFR2_NON_CANONICAL_NF_KB_PATHWAY
REACTOME_AUF1_HNRNP_D0_BINDS_AND_DESTABILIZES_MRNA
REACTOME_DECTIN_2_FAMILY
REACTOME_MITOTIC_SPINDLE_CHECKPOINT
REACTOME_RUNX1_REGULATES_TRANSCRIPTION_OF_GENES_INVOLVED_IN_DIFFER-ENTIATION_OF_HSCS
REACTOME_HOMOLOGOUS_DNA_PAIRING_AND_STRAND_EXCHANGE
REACTOME_CELLULAR_RESPONSE_TO_HYPOXIA
REACTOME_SYNTHESIS_OF_ACTIVE_UBIQUITIN_ROLES_OF_E1_AND_E2_ENZYMES
REACTOME_SIGNALING_BY_ERYTHROPOIETIN
PID_BCR_5PATHWAY
REACTOME_IRE1ALPHA_ACTIVATES_CHAPERONES
PID_FANCONI_PATHWAY
REACTOME_INTERLEUKIN_2_FAMILY_SIGNALING
REACTOME_AURKA_ACTIVATION_BY_TPX2
REACTOME_DEGRADATION_OF_AXIN
REACTOME_SURFACTANT_METABOLISM
REACTOME_TRANSCRIPTIONAL_REGULATION_BY_RUNX3
REACTOME_ORC1_REMOVAL_FROM_CHROMATIN
REACTOME_MRNA_CAPPING
BIOCARTA_CTLA4_PATHWAY
REACTOME_CYCLIN_A_CDK2_ASSOCIATED_EVENTS_AT_S_PHASE_ENTRY
PID_ARF_3PATHWAY
KEGG_HUNTINGTONS_DISEASE
REACTOME_ACTIVATION_OF_IRF3_IRF7_MEDIATED_BY_TBK1_IKK_EPSILON
REACTOME_NEGATIVE_REGULATION_OF_MAPK_PATHWAY
REACTOME_ANTIGEN_ACTIVATES_B_CELL_RECEPTOR_BCR_LEADING_TO_GENERA-TION_OF_SECOND_MESSENGERS
REACTOME_DECTIN_1_MEDIATED_NONCANONICAL_NF_KB_SIGNALING
REACTOME_SIGNALING_BY_NOTCH4
REACTOME_DAP12_INTERACTIONS
KEGG_PROTEASOME
KEGG_HOMOLOGOUS_RECOMBINATION

REACTOME_COSTIMULATION_BY_THE_CD28_FAMILY
REACTOME_ACTIVATION_OF_ATR_IN_RESPONSE_TO_REPLICATION_STRESS
BIOCARTA_TNFR2_PATHWAY
PID_CD8_TCR_DOWNSTREAM_PATHWAY
KEGG_INTESTINAL_IMMUNE_NETWORK_FOR_IGA_PRODUCTION
REACTOME_FCERI_MEDIATED_MAPK_ACTIVATION
REACTOME_CROSS_PRESENTATION_OF_SOLUBLE_EXOGENOUS_ANTIGENS_ENDOSOMES
BIOCARTA_STRESS_PATHWAY
REACTOME_ABORTIVE_ELONGATION_OF_HIV_1_TRANSCRIPT_IN_THE_ABSENCE_OF_TAT
REACTOME_TELOMERE_C_STRAND_LAGGING_STRAND_SYNTHESIS
REACTOME_DUAL_INCISION_IN_GG_NER
REACTOME_PROGRAMMED_CELL_DEATH
REACTOME_INTERLEUKIN_1_SIGNALING
PID_MET_PATHWAY
REACTOME_HATS_ACETYLATE_HISTONES
KEGG_CELL_CYCLE
REACTOME_ESTROGEN_DEPENDENT_GENE_EXPRESSION
REACTOME_PROCESSING_OF_CAPPED_INTRONLESS_PRE_MRNA
REACTOME_HIV_TRANSCRIPTION_INITIATION
PID_IL8_CXCR2_PATHWAY
PID_CDC42_PATHWAY
REACTOME_TBC_RABGAPS
KEGG_DNA_REPLICATION
REACTOME_E3_UBIQUITIN_LIGASES_UBIQUITINATE_TARGET_PROTEINS
REACTOME_THE_ROLE_OF_NEF_IN_HIV_1_REPLICATION_AND_DISEASE_PATHOGENESIS
REACTOME_RESPONSE_OF_MTB_TO_PHAGOCYTOSIS
REACTOME_DISEASES_ASSOCIATED_WITH_N_GLYCOSYLATION_OF_PROTEINS
REACTOME_IMMUNOREGULATORY_INTERACTIONS_BETWEEN_A_LYMPHOID_AND_A_NON_LYMPHOID_CELL
PID_AR_TF_PATHWAY
REACTOME_HDACS_DEACETYLATE_HISTONES
REACTOME_PCNA_DEPENDENT_LONG_PATCH_BASE_EXCISION_REPAIR
REACTOME_GPVI_MEDIATED_ACTIVATION_CASCADE
BIOCARTA_TNFR1_PATHWAY
PID_NFKAPPAB_ATYPICAL_PATHWAY
BIOCARTA_TCR_PATHWAY
PID_GMCSF_PATHWAY
REACTOME_RECOGNITION_OF_DNA_DAMAGE_BY_PCNA_CONTAINING_REPLICATION_COMPLEX
REACTOME_DAP12_SIGNALING
REACTOME_RESOLUTION_OF_AP_SITES_VIA_THE_MULTIPLE_NUCLEOTIDE_PATCH_REPLACEMENT_PATHWAY
KEGG_PROTEIN_EXPORT

BIOCARTA_BCR_PATHWAY
REACTOME_CELLULAR_SENESCENCE
REACTOME_MYD88_INDEPENDENT_TLR4_CASCADE
REACTOME_TP53_REGULATES_TRANSCRIPTION_OF_CELL_CYCLE_GENES
REACTOME_HDR_THROUGH_SINGLE_STRAND_ANNEALING_SSA
REACTOME_TRANSCRIPTIONAL_REGULATION_BY_RUNX1
REACTOME_DISEASES_OF_IMMUNE_SYSTEM
BIOCARTA_HIVNEF_PATHWAY
REACTOME_REGULATION_OF_RAS_BY_GAPS
KEGG_MISMATCH_REPAIR
REACTOME_MISMATCH_REPAIR
BIOCARTA_IL2RB_PATHWAY
REACTOME_INTERLEUKIN_RECEPTOR_SHC_SIGNALING
REACTOME_REGULATION_OF_HSF1_MEDIATED_HEAT_SHOCK_RESPONSE
SA_B_CELL_RECEPTOR_COMPLEXES
REACTOME_COPI_MEDIATED_ANTEROGRADE_TRANSPORT
REACTOME_GAP_FILLING_DNA_REPAIR_SYNTHESIS_AND_LIGATION_IN_GG_NER
REACTOME_ENDOSOMAL_SORTING_COMPLEX_REQUIRED_FOR_TRANSPORT_ESCRT
REACTOME_ABC_FAMILY_PROTEINS_MEDIATED_TRANSPORT
KEGG_N_GLYCAN_BIOSYNTHESIS
REACTOME_SCF_SKP2_MEDIATED_DEGRADATION_OF_P27_P21
REACTOME_FORMATION_OF_INCISION_COMPLEX_IN_GG_NER
PID_IL3_PATHWAY
REACTOME_NEUTROPHIL_DEGRANULATION
BIOCARTA_RAS_PATHWAY
BIOCARTA_GRANULOCYTES_PATHWAY
REACTOME_TRANSLESION_SYNTHESIS_BY_POLH
REACTOME_N_GLYCAN_TRIMMING_IN_THE_ER_AND_CALNEXIN_CALRETICULIN_CYCLE
REACTOME_RNA_POLYMERASE_I_TRANSCRIPTION_INITIATION
BIOCARTA_IL2_PATHWAY
PID_TOLL_ENDOGENOUS_PATHWAY
REACTOME_APC_CDC20_MEDIATED_DEGRADATION_OF_NEK2A
REACTOME_DNA_DAMAGE_RECOGNITION_IN_GG_NER
REACTOME_HDMS_DEMETHYLATE_HISTONES
REACTOME_COPII_MEDIATED_VESICLE_TRANSPORT
REACTOME_RNA_POLYMERASE_I_TRANSCRIPTION_TERMINATION
PID_FCER1_PATHWAY
BIOCARTA_RELA_PATHWAY
REACTOME_CD209_DC_SIGN_SIGNALING
REACTOME_REGULATION_OF_TP53_ACTIVITY_THROUGH_METHYLATION
KEGG_HEMATOPOIETIC_CELL_LINEAGE
REACTOME_RAB_REGULATION_OF_TRAFFICKING
REACTOME_ABC_TRANSPORTER_DISORDERS
PID_EPHA2_FWD_PATHWAY

KEGG_LYSOSOME
REACTOME_EXTENSION_OF_TELOMERES
REACTOME_RESOLUTION_OF_D_LOOP_STRUCTURES
